# Reluctance in international politics: A conceptualization

**DOI:** 10.1177/1354066116653665

**Published:** 2016-06-24

**Authors:** Sandra Destradi

**Affiliations:** Helmut Schmidt University / University of the Federal Armed Forces Hamburg and GIGA German Institute of Global and Area Studies, Germany

**Keywords:** Foreign policy, governance, International Relations, power, reluctance, rising powers

## Abstract

Contemporary rising powers have often pursued a hesitant and ambiguous foreign-policy and have belied the expectations of potential followers and established powers who would want them to engage more actively in global and regional governance. The existing analytical toolbox of International Relations does not offer suitable concepts to make sense of the widespread phenomenon of states that pursue hesitant, inconsistent courses of action and do not bring to bear their power resources to coherently manage international crises that potentially affect them. A notion that is frequently employed to describe this peculiar type of foreign policy is that of ‘reluctance’, but this concept has not been systematically defined, discussed or theorized. This article aims to introduce the concept of reluctance into the field of International Relations. It develops a conceptualization of reluctance by identifying the concept’s semantic field and discussing how reluctance relates to the similar but distinct notions of exceptionalism, isolationism, under-aggression and under-balancing (concept reconstruction); on that basis, the article outlines the constitutive dimensions of reluctance — hesitation and recalcitrance — and their operationalization (concept building). Several illustrative cases of (non-)reluctant rising powers are used to exemplify the concept structure and to show the analytical usefulness of the concept of reluctance, which refers to a distinct set of phenomena that are not addressed by other concepts in International Relations. An application of the concept allows us to identify policy shifts and differences across issue areas, as well as open up avenues for further research.

## Introduction

While the world seems to have become increasingly conflict-ridden and unpredictable, both established and rising powers frequently do not live up to the expectations of those who want them to provide leadership, order, governance and the management of international crises (Schweller, 2014). While the US has been increasingly preoccupied with domestic problems over the past years, rising powers like India, Brazil, China or South Africa have not displayed a readiness to step into the fray and contribute to the provision of order beyond a certain point. The 2015 climate summit of Paris was successfully concluded with an agreement, but rising powers continue to have strong reservations about binding targets for greenhouse gas emissions. In 2011, rising powers did not veto United Nations Security Council (UNSC) Resolution 1973 on the protection of civilians in Libya, but they did not engage in stabilization efforts in the Middle East and North Africa either. Furthermore, even in their own regions, where they have a long history of engagement and where their predominance is unequivocal, rising powers have pursued ambivalent and indecisive foreign policies. Brazil, for example, has at times been proactive in dealing with South American countries, for instance, by promoting the Southern Common Market (MERCOSUR), but it has been unwilling to delegate extensive decision-making powers to regional organizations and to ‘become the regional paymaster providing collective public goods such as credit, aid or security’ (Merke, 2015: 184). South Africa’s approach to Africa has similarly been characterized as full of ‘ambiguities and contradictions’ (Alden and Le Pere, 2009: 145), and India has pursued an ambivalent and reactive policy in South Asia, merely responding to initiatives developed by others despite its clear power preponderance (Ganguly, 2003). While Germany emerged as the regional power in Europe in the context of the Eurozone crisis (Bulmer and Paterson, 2013), several observers have noted that ‘[l]eadership from Berlin has been hesitant’ and plagued by a ‘capacity–expectations gap’ (Bulmer, 2014: 1245). Even China, which has been rather assertive in its own region, has been described as a ‘conflicted’ state (Shambaugh, 2011) lacking a clear grand strategy (Schweller, 2014: 69).

A notion that is frequently used in the literature to characterize this attitude of rising powers is *reluctance*. The *Munich Security Report 2015* (Bunde and Oroz, 2015), a brief publication associated with the Munich Security Conference, is entitled *Collapsing Orders, Reluctant Guardians?*: it argues that the collapse of international order has itself been ‘both a driver and an effect of the increasing reluctance of its traditional guardians’ (Bunde and Oroz, 2015: 22), like the US, as well as of potential new guardians, the rising powers. India and Germany have both been dubbed ‘reluctant hegemons’ with regard to their regions (Mitra, 2003; Paterson, 2011) and South Africa has been termed a ‘reluctant leader’ in Africa (Esterhuyse, 2010).

We therefore have a term to describe the peculiar type of foreign policy that rising powers (and often established powers as well) are pursuing — *reluctance*. However, we lack a systematic understanding of what reluctance means. The existing analytical toolbox of International Relations (IR) does not offer suitable concepts to make sense of the widespread phenomenon of powerful or rising states that pursue inconsistent, confusing courses of action and do not bring to bear their power resources to coherently manage international crises that potentially affect them. As will be discussed later in greater detail, this issue is implicitly addressed in large sections of the literature – for example, through the concepts of exceptionalism, isolationism, under-aggression and under-balancing. However, these notions refer to distinct sets of phenomena that have only a few commonalities with reluctance, but are not able to account for the phenomenon of reluctance itself.

Against this backdrop, this contribution aims to introduce reluctance into the study of international politics. It focuses on developing a thorough conceptualization of reluctance, since the casual, unspecific usage of the term in the existing literature is not of much use beyond description. By contrast, if appropriately conceptualized, ‘reluctance’ can yield analytical benefits by helping us to make sense of the widespread indecisiveness and responsibility shirking that seem to have become distinctive features of contemporary international politics.

The conceptualization of reluctance builds upon and combines different approaches to concept reconstruction and concept building, which are briefly discussed in the next section. The actual conceptualization exercise proceeds as follows. First, based on a qualitative content analysis of selected IR literature that explicitly uses the notion of reluctance, I inductively identify the key issues usually associated with this term. This helps as a first approach to delineating the broader semantic field of reluctance, and thereby contributes to concept reconstruction, as suggested by Sartori (1984: 41–50). Based on this broader semantic field, in a second step, I move on to discussing the related but distinct notions of exceptionalism, isolationism, under-aggression and under-balancing, and I highlight why we need reluctance as an additional concept to make sense of a distinct set of phenomena. Based on the insights gained from situating the concept of reluctance in the existing IR literature, I proceed with the actual concept-building exercise, which follows the guidelines outlined by Goertz (2006) in his work on social science concepts. I therefore: discuss the negative poles of reluctance — that is, what reluctance is not; develop two core ‘secondary’, constitutive dimensions of reluctance; and operationalize these two dimensions, developing indicators for empirical analysis. In a nutshell, I conceive of reluctance as a specific way or style of doing foreign policy that involves a hesitant attitude and a certain recalcitrance about conforming to the expectations articulated by others. To illustrate the analytical value of my conceptualization of reluctance, I finally apply the concept to an assessment of a range of cases of (non-)reluctant rising powers. I conclude by discussing how this more precise conceptualization of reluctance as a specific way of doing foreign policy can help us make sense of the policies of rising powers, and I identify a range of questions to be addressed by future research.

## Mapping the field: Reconnecting concept reconstruction and concept building

While the importance of concepts in the social sciences cannot be underestimated, extensive reflection on the process of defining and clarifying concepts remains rare, with notable exceptions (e.g. Collier et al., 2012; Goertz, 2006; Sartori, 1970, 1975, 1984). Among the few studies that explicitly deal with the issue of concept formation in the social sciences, there is disagreement on the first step to take. Sartori suggests that this first step should always be ‘concept reconstruction’, which amounts to tracing the use of the concept in previous works in order to assess how others have defined it and to extract and systematize underlying characteristics (Sartori, 1984: 41–50). As a subsequent step, Sartori recommends ‘allocation of the term’, that is, the choice of a specific word to be associated with the concept one intends to study. To do this, one needs to relate the term that designates the concept to its semantic field to make sure that reconceptualization does not lead to a loss of meaning of other terms, or to an increase in ambiguity instead of greater clarity (Sartori, 1984: 51–53).^1^ By semantic field, Sartori (1984: 52) refers to ‘a clustering of terms such that each of its component elements interacts with all the others, and (as with all systems) is altered by any alteration of the others’. Only after concept reconstruction and allocation of the term can we proceed to the main step of reconceptualization or concept building, according to Sartori. In a similar fashion, Adcock and Collier (2001: 531) argue that conceptualization — that is, the formulation of a systematized concept — must be based on an assessment of what they call the ‘background concept’: the existing ‘broad constellation of meanings and understandings associated with a given concept’.

Much more than Sartori, Goertz (2006: 4) highlights that thinking about concepts involves going well beyond semantics — it implies carrying out a ‘theoretical and empirical analysis of the object or the phenomenon’ that is being conceptualized. Correspondingly, Goertz (2006: 5) thinks of concepts: in *ontological* terms, since conceptualizations imply focusing on what constitutes a phenomenon; in *causal* terms, since the central dimensions of concepts have causal powers, which, in turn, shape theories that employ these concepts; and in *realist* terms, since concepts always relate to empirical phenomena. To adequately address these aspects, it is important to address the structure of concepts and the relationships between the different dimensions and levels within concepts in the concept-building exercise. According to Goertz (2006: 27, emphasis added), developing a concept amounts to ‘*deciding* what is important about an entity’. However, how can we make this essential decision? In other words, how do we know what *is* important about an entity? In order to avoid at least some of the arbitrariness that might be associated with starting the concept-building exercise without appropriate groundwork, it is useful to start with concept reconstruction, as suggested by Sartori — or to assess the background concept, as Adcock and Collier (2001: 531) put it, or to apply the ‘context guideline’, according to Goertz and Mazur (2008: 19–20). The need to build upon previous uses of a concept seems particularly compelling for notions like reluctance, which are already frequently employed in the literature but in a confused and unspecific manner — that is, when concept building is used to clarify the meaning of an existing term.

### Concept reconstruction: The broader semantic field

As a first step, it is therefore useful to map the field, that is, to analyse existing literature that uses the concept of reluctance in order to find out how the concept is defined, what notions and types of behaviour are usually associated with it, and what its ‘set of associated, neighboring terms’ (Sartori, 1984: 52) is. While the notion of reluctance is not specific to IR or to political science and is, indeed, frequently used in a range of fields, from medicine to sociology and psychology, to analyse specific forms of behaviour, a striking commonality across disciplines is an absence of definitions or sophisticated operationalizations of reluctance. For example, clinical studies that deal with patients’ reluctance to take preventive medication, to undergo preventive tests or to seek treatment either simply equate reluctance with a choice not to do something (Quaid and Morris, 1993) or, in a slightly more sophisticated manner, associate reluctance with a general *resistance* to taking medication (Port et al., 2001) or with the notion of not seeing a doctor despite knowing one should do so (Meltzer et al., 2000). Studies in the field of sociology simply operationalize reluctance by looking at different degrees of stated *(un)willingness* (Bielby and Bielby, 1992). Moreover, to mention another example, in a study on reluctance to communicate undesirable information, reluctance is operationalized as a *time lag* in the transmission of information or as the transmission of *incomplete information* (Rosen and Tesser, 1970).

**Figure 1.**
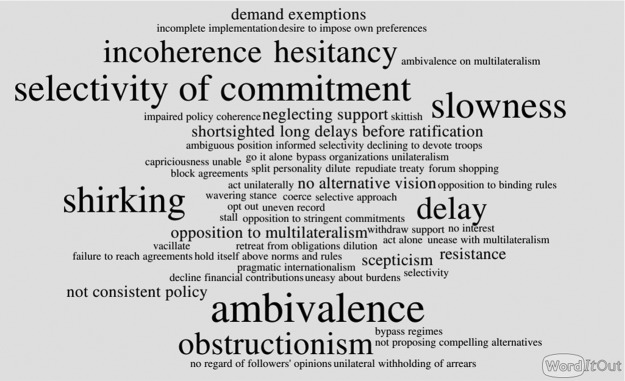
Notions associated with reluctance in international politics. This figure was created using worditout.com.

In studies that focus on the reluctance of states in foreign policy, that is, in the fields of IR and history, we observe a similar lack of definitions. Most studies that prominently mention reluctance in their titles do not explicitly discuss the concept, and, in the case of books, do not even include the term in the index (e.g. Dueck, 2006; Fehl, 2012; Haass, 1997; Lowe, 1967). Nevertheless, these studies address a specific type of state behaviour or a specific way of doing foreign policy that has distinct and identifiable characteristics. In order to gather more systematic information on how reluctance is understood in the field of IR, I have carried out a qualitative content analysis of selected studies that prominently use this term, proceeding inductively, that is, approaching the text corpus without a predefined set of categories. I have explicitly avoided building my text corpus on works that just refer to the reluctance of rising powers in order to exclude circular reasoning while applying the concept of reluctance to the same group of states — rising powers — in later sections of this study. Most works in the field of IR that explicitly address ‘reluctance’ refer to the US or other great powers, probably because of the puzzling and paradoxical coexistence of resource abundance or ‘hegemony’ with hesitant foreign policies and responsibility shirking. However, the analysis also included works on smaller reluctant states (e.g. Gstöhl, 2002). The results of the analysis are displayed in Figure 1.^2^

Reluctance is usually associated, among other things, with a highly *ambivalent attitude*, *hesitant behaviour* and a *selective commitment*. For example, as Fehl (2012: 10) highlights, a reluctant US was the main proponent of an International Trade Organization after the Second World War but later stopped supporting the idea; it signed most human rights conventions but then did not ratify them during the Cold War. In the post-Cold War world, according to Haass (1997), the US has become a ‘reluctant sheriff’, struggling with the costs of providing order and with decreasing domestic interest in and consensus on foreign-policy issues. This reluctance is associated with ad hoc, short-term approaches and with a lack of ‘clarity and soundness of purpose’ (Haass, 1997: 3).^3^ Schweller (2014: 6) similarly sees a danger in the current attitude of the US, which risks becoming an ‘elephant on the sidelines, a potential but reluctant hegemon unwilling to lead’. An analysis of Norway’s, Sweden’s and Switzerland’s approaches to European integration identifies reluctance with scepticism towards integration. A preference for more limited forms of integration and the adoption of hesitant policies are seen as distinctive of this reluctant attitude (Gstöhl, 2002: 3–4). Related to ambivalence, hesitation and selectivity of commitment is a high degree of *incoherence* in a reluctant country’s policies — as Patrick (2002: 5) puts it, Washington ‘has often seemed skittish about committing itself to proposed international legal regimes’, for example, on the International Criminal Court (ICC) or on human rights conventions. Similarly, Britain in the second half of the 19th century has been termed a ‘reluctant imperialist’, with reluctance amounting to caution and aloofness from European affairs (Lowe, 1967: 9) and, interestingly, to a certain ‘amateurishness’ in foreign policymaking (Lowe, 1967: 13). Other elements associated with reluctance are an *obstructionism towards others’ initiatives* and a certain *slowness* in implementing policies, with delays and sometimes even the adoption of ‘time-buying’ tactics. For example, the US Senate during the 1990s ‘stalled, diluted, or defeated’ a range of multilateral initiatives in the field of control of weapons of mass destruction, with the most evident case being the rejection of the Comprehensive Test Ban Treaty (Patrick, 2002: 3).

In sum, an assessment of the broader semantic field of reluctance reveals that this notion is related to policies that are ambivalent and, at times, even obstructionist towards the initiatives of others, which involve shirking responsibility, a hesitant attitude, delays in implementation, selectivity of commitments and incoherence.

### Concept reconstruction: Related theoretical approaches

**Figure 2.**
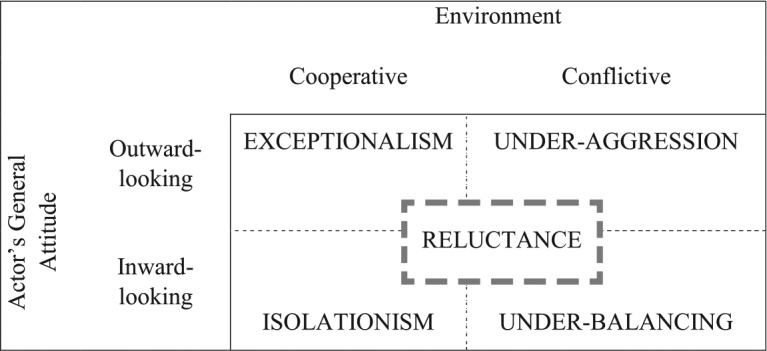
Concepts related to reluctance.

Based on the preceding assessment of the broader semantic field of reluctance, a further useful step in concept reconstruction consists in relating the concept to existing theoretical approaches in the field, in this case, in the subject area of IR. In particular, there are four notions that are explicitly or implicitly related to the concept of reluctance as it has developed from the analysis of the broader semantic field, and that describe different types of foreign policy that powerful or rising states can adopt. They are exceptionalism, isolationism, under-aggression and under-balancing. Figure 2 provides an overview of these concepts and an illustration of how reluctance relates to them, with the dashed lines indicating that the concepts are not just closely interrelated, but sometimes even overlapping. The four concepts, which will be discussed in greater detail in the following sections, can be ordered according to the cooperative versus conflictive nature of the environment, as well as to the general attitude of the actor, which can be more or less outward- versus inward-looking.

The concept of *exceptionalism* refers to a body of literature that studies the foreign policy of states that have a particular sense of entitlement or a perception of being exceptional and therefore not subject to the rules and constraints binding other states. This leads, on the one hand, to policies aimed at ‘liberating’ others in the name of some messianic belief or some special responsibility (Holsti, 2010) — a feature that is barely related to reluctance. With reference to the US, underlying this approach to exceptionalism is the notion that an exceptional country ‘should be exempted from the international rules that bind other nations’ (Halperin and Boyer, 2007: 2). On the other hand, therefore, exceptionalism entails efforts to achieve freedom of action by shedding the burden of cooperation in multilateral institutions and regimes (Halperin and Boyer, 2007; Holsti, 2010). Rising powers are also frequently characterized as actors that shirk responsibilities in international settings, ‘want the privileges of power but are unwilling to pay for them by contributing to global governance’ (Schweller and Pu, 2011: 42) and are not willing to lead or to bear the costs of public good provision. Therefore, exceptionalism is related to ambivalence and a general unwillingness to commit, which are important elements generally associated with the notion of reluctance. Exceptionalism is placed on the side of ‘outward-looking’ attitudes in Figure 2 as it refers not only to a passive and self-referential unwillingness to get entangled (isolationism), but also to a more general recalcitrance about actively promoting multilateral cooperation or collective action. The potential decisiveness of exceptionalist powers in bilateral settings, in turn, distinguishes exceptionalism from reluctance, which is usually associated with hesitant, indecisive action.

The literature on *isolationism* focuses on powerful countries that decide to pursue a ‘minimalist’ foreign policy characterized by limited goals, a high degree of restraint^4^ and a limited amount of resources devoted to foreign policy (Haass, 1997: 55). Isolationism does not only involve an unwillingness to use military force: isolationist countries prefer not to get engaged abroad at all, are clearly inward-looking and therefore limit the resources devoted to different instruments of foreign policy, including diplomacy and foreign aid (Haass, 1997: 57). Correspondingly, isolationism is mostly equated with a policy of non-entanglement and with abstention from commitments. Authors like Legro (2005: 51) prefer to talk about ‘separatism’, understood not as strict isolation, but as ‘nonengagement, aloofness, and detached unilateralism’. Among the typical examples of isolationist countries are Japan between the 18th century and the Meiji Restoration — a case of strict isolationism (Haass, 1997: 128–131) — and the US between the world wars, which was certainly not fully isolated, but refused to get engaged abroad and was characterized by strong isolationist ideas that were only slowly replaced by an internationalist approach (Haass, 1997: ch. 3).^5^ The notion of isolationism does not exclusively or necessarily apply to military interventions or conflictive settings. It can also mean that states do not make use of cooperative opportunities. The close relationship between the concepts of isolationism and reluctance is highlighted, for example, by Dueck (2006: 27), who explicitly equates a ‘preference for nonentanglement’ with reluctance. At the same time, there are differences between the two concepts. For example, the element of hesitation, which is mostly associated with reluctance, does not necessarily form part of an isolationist policy: consistent and resolute isolationism (Nordlinger, 1995: 9) has little in common with the indecisiveness that is typical of reluctance.

The literature on *under-aggression* or under-expansion focuses specifically on rising powers and tries to explain why an increase in power capabilities does not necessarily translate into an aggressive and expansionist foreign policy, as expected by offensive realism. Among the typical cases analysed is the ‘imperial understretch’ of the US after the Civil War, which Zakaria (1998), for example, explains with the weakness of US state structure. Dueck (2006: 2) relates the notion of under-expansionism to the concept of reluctance by asking why Americans have been ‘reluctant crusaders’: ‘crusaders in the promotion of a more liberal international order’ but ‘reluctant to admit the full costs of promoting this liberal international vision’. Recently, Meiser (2015) has added to the literature on US under-expansionism (or, in his words, restraint) by focusing, again, on why the rising US did not expand territorially between 1898 and 1941, with his explanation mostly focusing on domestic institutions and the path-dependency effects of restraint. Among the authors who look beyond the case of the US is Schweller, who investigates what he calls ‘the suboptimal reluctance to use force or to build up military power’ (Schweller, 2006: 105) on the part of most great powers in the 20th century. His contentious conclusion is that only fascist regimes could develop the ‘“ideologized” power politics for mass consumption’ (Schweller, 2006: 21) that are required for expansionism in the modern world and involve a substantial mobilization of the population. The notions of under-aggression and under-expansion therefore refer to policies of countries that are not necessarily inward-looking, but for different reasons do not realize their outward-looking potential in military terms. These notions relate to reluctance, but with a specific reference to conflictive policies, thereby excluding the more subtle nuances mostly associated with the concept of reluctance, which involves a whole array of indecisive, hesitant, flip-flopping policies. While most approaches explain under-aggression as an ‘unwillingness’ to expand, some also take into account the ‘inability’ or, more generally, the constraints faced by powerful countries. Among the main limitations of approaches that focus on under-aggression or restraint is that they necessarily have to work with counterfactuals to explain why something that would have been expected has *not* taken place (see, e.g., Meiser, 2015). By contrast, the notion of reluctance, if appropriately conceptualized and operationalized, refers to actual foreign policy.

Finally, a related notion is that of *under-balancing*: the attitude of countries that do not respond to threats in conflictive settings by mobilizing military power or by forging alliances (Schweller, 2006). Unlike under-aggression, this concept does not focus on a lack of initiative or on why states are ‘timid’ (Schweller, 2006: 20). Instead, it focuses on why states do not react to dangerous aggressors or to dangerous changes in relative power as expected by balance-of-power theory. It refers, therefore, to inward-looking policies, as outlined in Figure 2. Among the four concepts, under-balancing is probably most closely associated with reluctance. As Schweller (2006: 63) explicitly acknowledges it can take the form of ‘half measures, muddling through, and incoherent grand strategies’ and thus reflects the incoherence and ambivalence that is frequently associated with reluctance. However, under-balancing addresses only a very specific context for reluctant behaviour, namely, the situation of being threatened by another country — a situation in which reluctance is a ‘mistake’ (Schweller, 2006: 10) that potentially affects a state’s survival. Therefore, it does not refer to a broader spectrum of foreign-policy contexts and situations that go beyond the particular setting of being threatened. Moreover, focusing on under-balancing as a ‘mistake’ ignores cases in which buying time, preferring not to commit too heavily or shirking responsibility might be adequate ways of dealing with different types of pressure and expectations.

The concept of reluctance therefore touches upon each of the four concepts discussed earlier and yet differs from them in important respects. Each of them is ‘a generalizable behaviour, as opposed to a unique or individual occurrence, that shares certain distinguishing features, such that it forms a class of historical cases’ (Schweller, 2006: 16). The same holds for reluctance. What the four concepts of exceptionalism, isolationism, under-aggression and under-balancing share is the diffuse notion of *not* doing something that might be appropriate — of being forms of behaviour that do not conform to certain expectations. This notion of not fulfilling the expectations of others is an important element associated with reluctance. Expectations might either involve calls to adopt a different policy or exhortations to stop implementing certain policies, and both can be met with recalcitrance. At the same time, the concept of reluctance stands between exceptionalism, isolationism, under-aggression and under-balancing since it can refer to both inward- and outward-looking actors in both cooperative and conflictive settings. This implies that reluctance is not limited to one specific policy field. The question ‘Reluctance to do what?’ can therefore be answered in very different ways. Reluctance itself does not necessarily just refer to an unwillingness to lead or to pursue hegemonic policies, as suggested by studies that resort to notions like ‘reluctant hegemony’. Furthermore, it is not necessarily just ‘reluctance to do something’, but can also refer to a hesitant and recalcitrant approach with regard to *not* doing something or discontinuing existing policies. Reluctance therefore rather constitutes a peculiar *type* or *style* of foreign policy that can be found across issue areas and settings. We therefore need a better understanding of reluctance as a concept that refers to a distinct set of phenomena.

## Reluctance: Concept building

The previous identification of the broader semantic field of reluctance in IR and our discussion of related theoretical approaches has allowed us to obtain some insights into the ‘background concept’ (Adcock and Collier, 2001: 531) of reluctance. This is a useful starting point: ‘concept reconstruction is a means whose ultimate purpose is to provide a cleaned-up basis for construction — that is, for the *formation* of concepts’ (Sartori, 1984: 50, emphasis in original). In other words, concept reconstruction helps us to grasp ‘what is important about an entity’ (Goertz, 2006: 27). On this basis, we can now proceed with concept building, and, to that end, we will follow the guidelines proposed by Goertz (2006) as this work provides important ideas on how to think about the structure of concepts.

### The negative poles

The first step in concept building consists in identifying the ‘basic level’ of a concept, that is, the concept ‘as used in theoretical propositions’ (Goertz, 2006: 6). In our case, the basic-level concept is *reluctance*. A useful way to sharpen our understanding of a basic-level concept entails thinking explicitly about the negative pole of that concept, that is, about what the concept is *not*. For the case of reluctance, there is not a single obvious negative pole. Within the broader idea of ‘non-reluctance’, however, we can think of two dimensions that constitute the opposite of reluctance on two different continuums: (1) determination; and (2) being responsive to demands made by others. *Determination* amounts to a resolute, decisive and consistent attitude, which involves the ability to make decisions without displaying much hesitation. The concept of determination therefore encompasses the opposite of the ambivalence, incoherence, hesitancy and slowness that characterize reluctance. By *responsiveness to others’ demands*, I understand reacting readily and sympathetically to appeals and requests by other actors, particularly by less powerful actors in a hierarchical setting like that existing between rising powers and smaller neighbouring states. Potential followers can ask the rising power to participate in common initiatives, to deliver public goods, to support a certain cause, to act in a less intrusive manner and many other things. A country that is responsive to such demands will not shirk responsibility, will not be unwilling to commit fully and will not display an obstructionist attitude towards the demands made by others — in other words, a responsive country will not be reluctant.

Determination and responsiveness therefore constitute different dimensions of the negative pole of reluctance. They can help us to clarify the actual ‘constitutive’ or ‘secondary-level’ dimensions of the concept of reluctance.^6^

### Constitutive dimensions and concept structure

**Figure 3.**
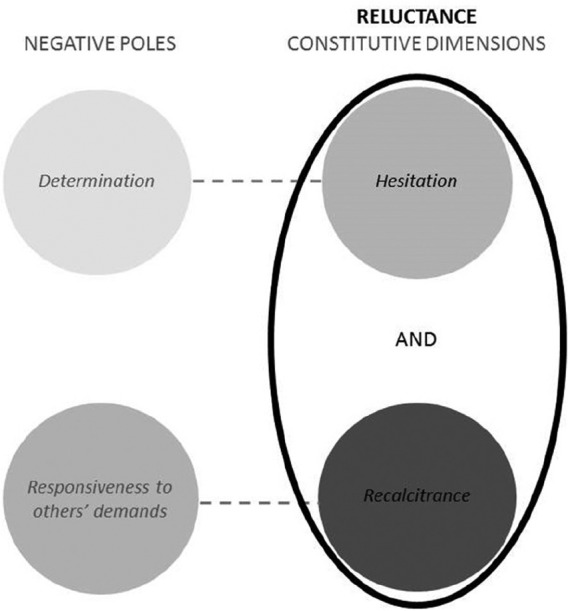
Negative poles and secondary/constitutive dimensions of reluctance.

As illustrated in Figure 3, reluctance has two constitutive dimensions: *hesitation* and *recalcitrance*. These dimensions are ‘constitutive’ in the sense that they tell us what the basic-level concept of reluctance consists of: ‘Concepts are theories about ontology: they are theories about the fundamental constitutive elements of a phenomenon’ (Goertz, 2006: 5). As such, concepts are not just important as constructs that help us to grasp empirical reality, but they also have an impact on theorizing and hypothesis testing. As a consequence, concepts and the ways in which they are constructed have huge implications for all the successive phases of a research project (think of the implications of conceptualization for case selection); elements of causality are often inherent in concepts, and need to be appropriately acknowledged.

Along a continuum entailing different degrees of resolve in an actor’s behaviour, *hesitation* is the polar opposite of determination. It involves an ambivalent, incoherent attitude. Reluctance is therefore much more than just under-aggression or under-expansionism. Nor does reluctance necessarily amount to the adoption of a ‘hands-off’ policy, that is, to inactivity. A consistent refusal to get involved in a military dispute, for example, is not reluctant behaviour understood as hesitation since it actually amounts to a clear and coherent policy course. For example, the fact that Japan and Germany were for several decades ‘reluctant to resort to the use of military force’ (Berger, 1996: 318) does not amount to reluctance as hesitation as understood in this study since their attitude was coherent. If, however, we observe contrasting and incoherent statements on the need to become active, or possibly even some engagement followed by backtracking, this will amount to reluctance.

*Recalcitrance* is the second core dimension of reluctance, and it is the opposite of being responsive to others’ demands — along a continuum referring to the degree of openness towards the wishes of others, especially of potential followers. It involves opposing the wishes articulated by others. For example, Fehl (2012: 4) highlights that the US displayed a ‘scepticism or outright opposition to multilateral treaty initiatives strongly favoured by European states’. The latter demanded a stronger commitment on the part of Washington, which, however, was recalcitrant — that is, unresponsive to the demands articulated by its European partners.

An essential step in building concepts is the clarification and discussion of their structure, which has huge consequences for their extension and intension (Sartori, 1970). In fact, depending on whether one adopts a necessary-and-sufficient-conditions structure or a ‘family resemblance’ structure that does not entail any necessary conditions (Goertz, 2006: 35–46), the number of secondary dimensions will have an impact on the categories of phenomena that are covered by the concept. The concept of reluctance as developed in this article follows a traditional necessary-and-sufficient-conditions structure: the two secondary dimensions of hesitation and recalcitrance are necessary and jointly sufficient to define reluctance — and are therefore connected by the logical operator AND. A family resemblance structure is not suitable for a conceptualization of reluctance since it implies that all conditions are sufficient, but that none is necessary. Adopting such a structure would have meant connecting hesitation and recalcitrance with the logical operator OR and arguing that either hesitation or recalcitrance would be sufficient for characterizing foreign policy as ‘reluctant’. However, in that case, the concept’s extension would grow indefinitely since recalcitrance — a lack of responsiveness to the demands made by others — is an almost omnipresent feature of foreign policy: states are continually confronted with expectations articulated by very different actors, be they other countries, international organizations, transnational non-governmental organizations (NGOs), their own public or other domestic actors. As these expectations and pressures will, in most cases, be contradictory, given the very different structural positions and interests of the actors articulating them, any foreign-policy decision will invariably ignore or reject the demands made by some of these actors. Therefore, if recalcitrance were a sufficient condition to conceptualize reluctance, any foreign-policy decision could be considered ‘reluctant’ and the concept itself would take a very different connotation as compared to its usage identified through concept reconstruction. Recalcitrance alone cannot therefore count as a sufficient condition to define reluctance: it needs to be paired with hesitation. Similarly, hesitation itself is not identical with reluctance because it does not automatically entail a lack of responsiveness vis-a-vis the expectations articulated by others — even though incoherent and contradictory statements and policies will usually disappoint at least some expectations. By conceptualizing foreign-policy reluctance as constituted by the necessary and jointly sufficient conditions of hesitation and recalcitrance, we avoid the ambiguities inherent in much of the literature, which amplify the concept’s extension by treating reluctance in a loose and unspecified manner.

These thoughts on the concept structure of reluctance have theoretical implications. For example, the inclusion of recalcitrance in the concept will have an impact on the development of a theory of reluctance in international politics. The simple observation of an indecisive, flip-flopping foreign policy on the part of a government could, in principle, be easily termed ‘reluctance’, but the inclusion of the element of recalcitrance, with its refusal to conform to the expectations of other actors, has important consequences. If we focus on the reluctance displayed by rising powers in their own regions and if we take the foreign policy of these countries as an independent variable to explain, for example, variations in regional cooperation, then recalcitrance matters. In fact, a recalcitrant attitude vis-a-vis the wishes articulated by smaller regional countries will most likely lead to disillusionment and disaffection among them, and thus contribute to hampering regional cooperation. For example, India has long been recalcitrant in its approach to its smaller neighbours in South Asia, refusing to make concessions on matters like trade. This has fuelled the suspicions of small countries of New Delhi and has induced them to see China as an attractive alternative partner — with disastrous consequences for regional cooperation in South Asia (Destradi, 2012). On a different note, if we take reluctance as a dependent variable, we might hypothesize that it can emerge as a consequence of the effort to manage competing expectations articulated by different actors at different levels of analysis — be they great powers at the global level, neighbouring states or domestic actors. Recalcitrance, understood as an unwillingness or impossibility to respond to all of these expectations, would then reflect the dilemma of states in which different domestic actors have different priorities, also concerning the accommodation of external actors’ expectations. Reluctance might therefore be considered an outcome of the political process of accommodating and mediating between different expectations.

## Operationalization

The third level of concepts is constituted by indicators to be applied in the analysis of empirical phenomena. An operationalization of reluctance in terms of hesitation and recalcitrance has the advantage of allowing us to avoid counterfactual arguments like those used in publications that refer to the untapped leadership potential of ‘reluctant’ powers (see, e.g., publications on Germany as a reluctant hegemon or, more generally, on Germany’s role in the Eurozone crisis (e.g. Jones, 2010: 26; *The Economist*, 2013)). In fact, those usages of the term ‘reluctance’ imply that something has not been done — for example, that Germany has not taken over a leadership role in Europe. Like the aforementioned studies on ‘under-aggression’, which use counterfactuals to explain why something that would have been expected (‘aggression’) has *not* taken place (Meiser, 2015), most works that refer to ‘reluctance’ vaguely associate this notion with a *lack* of leadership or of purpose. From such a perspective, reluctance would need to be thought of in terms of a counterfactual, that is, of what would have happened if an actor had not been reluctant — an approach that, of course, bears analytical difficulties. By contrast, this article develops indicators of hesitation and recalcitrance that refer to observable foreign-policy behaviour and to expectations explicitly articulated by other actors. Thereby, these indicators allow us to identify reluctance on the basis of actual foreign policy and to avoid counterfactual reasoning.^7^

Hesitation can be identified by the following indicators:

- Lack of initiative: with a particular focus on powerful countries such as rising powers, this indicator implies that smaller states develop and implement suggestions and solutions on a specific issue or crisis that is relevant not just to them, but also to the rising power. The powerful actor, therefore, free-rides on that initiative by not contributing resources proportionate to its weight and does not come up with its own policies and solutions. This indicator of hesitation refers to a passive policy as a powerful country stands on the sidelines and lets other actors take the lead.
- Delaying: hesitation can take a more explicit form if the dominant country buys time and does not stick to a previously agreed time frame, thereby postponing important decisions in dealing with a specific issue or crisis.^8^
- Flip-flopping in statements and/or policies: this is the most evident form of hesitation. It can be observed if the statements on a specific issue made by members of the executive or other official government representatives are not consistent over time, but change frequently or suddenly, or if policies on that issue are not coherent, but keep changing rapidly.^9^ Flip-flopping is also associated with contradictions in statements or policies among different representatives of the same government on a specific issue.

Recalcitrance, which is a lack of responsiveness towards the demands made by others, can be assessed through the following indicators:

- Ignoring requests made by others: the government of the dominant country does not react to calls made by other actors and its policies do not reflect the preferences articulated by them.
- Rejecting requests: the government of the dominant country explicitly refuses to comply with the wishes articulated by other actors.
- Obstructing others’ initiatives: the government of the dominant country hampers others’ activities. This does not necessarily happen through an explicit veto or other formal procedures, but can take place informally, for example, in the context of multilateral decision-making processes in which smaller countries will tend to conform to the preferences of a powerful actor.

**Figure 4.**
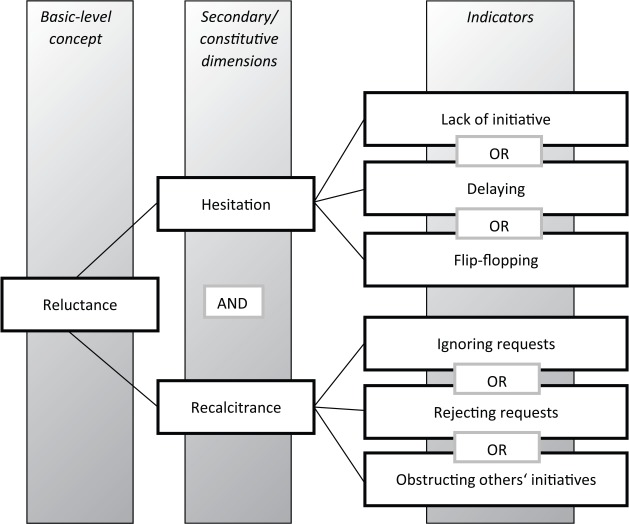
The concept structure of reluctance.

Among the indicators of both hesitation and recalcitrance (see Figure 4), there is a logical OR, which implies that for each dimension, it is sufficient to observe at least one of the three indicators in order to classify foreign policy as hesitant or recalcitrant, but that more than one indicator can be observed at the same time. For example, a hesitant country can both display a lack of initiative in the management of serious crises and, at the same time, delay initiatives proposed by others. Similarly, a recalcitrant country can both ignore requests made by others and, at the same time, hamper their initiatives.

Importantly, both hesitation and recalcitrance can occur to different degrees, implying that each of these dimensions entails a continuum. In his work on social science concepts, Goertz (2006: 34) highlights the usefulness of treating ‘all concepts as continuous’.^10^ This allows us to reduce measurement error by acknowledging that particular cases can lie at the weaker or the stronger end of a conceptual continuum; it thereby also helps to avoid theorizing on the basis of very special cases (Goertz, 2006). Relatedly, Goertz (2006) argues in favour of an ontology that explicitly acknowledges ‘borderline cases’ or ‘grey zones’. Grey zones include cases at the fuzzy border between positive cases, which correspond to a certain concept (in our study, cases of reluctance), and negative cases (in our study, cases of non-reluctance). Often, grey zones involve ‘transitions’: for example, Goertz (2006) mentions democratic transition as a typical grey zone between democracy and autocracy. The existence of such grey zones needs to be ‘openly confront[ed]’ (Goertz and Mazur, 2008: 30) since it has huge implications for both measurement and case selection.^11^ In the following paragraphs, I will discuss the notions of a continuous concept and of grey zones with reference to reluctance.

**Figure 5.**
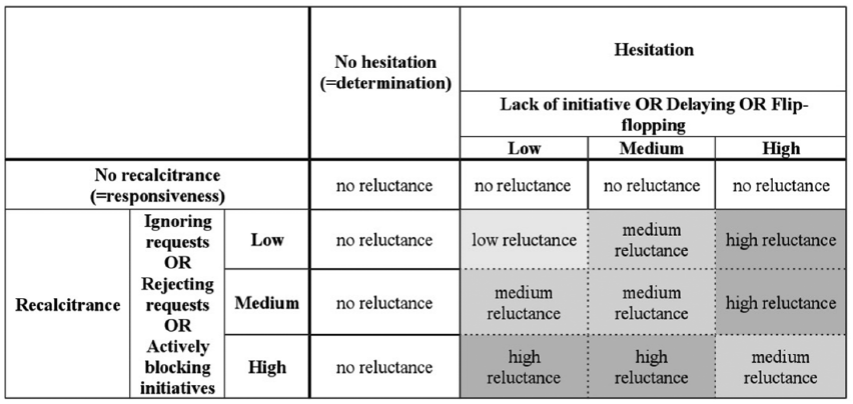
Intensity of reluctance.

The concept of reluctance is continuous since both its secondary dimensions — hesitation and recalcitrance — can occur to different degrees. The indicators of recalcitrance are arrayed along a continuum ranging from a mere ignoring of requests to the rejection of requests and the active obstructionism of initiatives promoted by other actors. Depending on the combination of these indicators, on the frequency with which they appear and on the salience of the issues on which a state is recalcitrant, we can classify recalcitrance as low, medium or high, with an appropriate weighting to be applied in specific empirical analyses. The same is true for hesitation, where a lack of initiative is a less explicit form of hesitation as compared to the delaying of decisions, while flip-flopping is the most evident type of hesitant behaviour. These different intensities and the possible different combinations of lack of initiative, delaying and flip-flopping can therefore lead to varying degrees of hesitation (see Figure 5). For example, a country whose foreign policy is strongly flip-flopping in crucial decision processes will obviously count as more reluctant than a country whose foreign policy displays little flip-flopping and strong initiatives, but some delays in the implementation of policies. As illustrated in Figure 5, the combination of indicators of hesitation and recalcitrance leads to the identification of different degrees of reluctant behaviour. This more fine-grained assessment of reluctance, which goes beyond a mere dichotomous understanding of the concept, can prove helpful in the assessment of variation in foreign-policy reluctance. Moreover, it will prove useful in tracing processes of policy change by highlighting shifts in the intensity of reluctance.

Table 1 also highlights that hesitation and recalcitrance are both necessary conditions for reluctance. That is, a recalcitrant but determined (non-hesitant) attitude — for example, a coherent refusal to comply with smaller regional countries’ wishes — would not amount to reluctance. Similarly, a hesitant but responsive attitude would not correspond to reluctance: while it might be hard for an actor to be fully responsive while pursuing an entirely incoherent, flip-flopping policy, a delayed reaction to the wishes of others or conforming to their wishes while not developing initiatives would not qualify as reluctance.

The ‘grey zones’ of reluctance include cases of transition between reluctant and non-reluctant policies. Two interesting grey zones emerge at the upper end of the continuum, that is, in cases of *extreme* recalcitrance or hesitation. If an actor is hesitant and extremely recalcitrant, this recalcitrance might become so strong as to prevail over hesitation: the blocking of others’ initiatives will become more and more determined, up to the point of leaving hesitation behind and becoming a coherent and consistent policy. Similarly, if recalcitrance is combined with extreme hesitation, we have another ‘grey zone’: indecisiveness can be so strong that the reluctant actor becomes unable to make a choice and gets temporarily ‘paralysed’. The transition towards non-reluctance will be completed when the paralysis turns into consistent inaction since that very inaction would amount to a determined attitude. An empirical illustration of one of the grey zones will be provided later in the discussion of the European Union’s (EU’s) approach to the Libya crisis in 2011. Another particular set of cases are those in which an actor is both highly recalcitrant (i.e. rather openly hampering the initiatives promoted by others) and, at the same time, highly hesitant. In this case, we can expect the two dimensions of hesitation and recalcitrance to partially offset each other, so that the outcome will be a comparatively more moderate form of reluctance (as illustrated in the bottom right field in Figure 5).

## An application: Reluctant rising powers?

As was mentioned in the introductory section, reluctance is frequently associated with the foreign-policy attitude of rising powers, but this term is often used in a rather loose and unspecified manner. An application of the concept of reluctance developed in this article can provide some clarity. Rising powers can be defined as countries that display ‘expanding economic prowess, a high degree of political power and military potential and the capability to play an influential role in global politics’ (Cooper and Flemes, 2013: 946).^12^ While most authors agree on considering China, India, Brazil and South Africa as rising powers, developments like the growing international visibility of Germany or the declining economic growth of Brazil remind us that membership in the club of rising powers can change. Russia, while being a member of the BRICS (Brazil, Russia, India, China and South Africa), does not fulfil some of the criteria mentioned earlier, particularly the one on expanding economic prowess, and should therefore be considered an ‘outlier’ (Hurrell, 2006: 2) and a declining power (Cooper and Flemes, 2013: 945).

In the following sections, I will proceed by looking at the different combinations of the two constitutive dimensions of reluctance and their different intensities, as illustrated in Figure 5. The selection of cases is driven by illustration purposes.

First of all, if a foreign policy displays *both determination and responsiveness*, it will not be reluctant. Among rising powers, the one that has generally been closest to non-reluctance in dealing with its region is Brazil. Since the late 1990s and up to the ‘Lula’ da Silva presidency, Brazil had developed a proactive and constructive approach towards South America. During those years, the Brazilian government dealt with some of the most serious regional crises: in a rather decisive manner, it prevented coups in Paraguay (1997) and Venezuela (2002), it mediated in the territorial dispute between Ecuador and Peru (1995–1998), and it led the UN Stabilization Mission in Haiti (MINUSTAH) (Spektor, 2010: 193). In the field of security, therefore, Brazil took the initiative, did not delay and was coherent in its policies. At the same time, it was mostly responsive to the demands of fellow South American countries, even though suspicions towards Brazil’s ambitious discourse of ‘non-indifference’ (Spektor, 2010: 194) towards the region remained in place among Brazil’s neighbours (Malamud, 2011). By contrast, when it comes to the promotion of regional integration, Brazil displayed a certain degree of recalcitrance as it ‘responded only selectively to calls for deepening regional institutions’ (Spektor, 2010: 195). While Brazil was the driving force in the creation of MERCOSUR and of what later became the Union of South American Nations (UNASUR), during Lula’s second presidential term and under Dilma Rousseff, its approach became more hesitant. Some degree of flip-flopping was revealed by Brazil’s ‘simultaneous rhetoric of solidarity and unwillingness to build robust regional multilateral institutions’ (Burges, 2015: 195) and by a coexistence of a cooperative and multilateralist rhetoric and an increasing assertiveness and preference for bilateralism (Burges, 2015: 201–102). The case of Brazil, which in the 1990s–2000s was not reluctant on security issues but has displayed an increasing reluctance in the field of regional integration in recent years, reveals possible discrepancies in reluctance across issue areas.

A historical comparison with Brazil’s policies in the 1960s and 1970s, in turn, provides an interesting contrast and an illustration of policies that are *recalcitrant but not hesitant* — and therefore non-reluctant. After the military coup of 1964, Brazil was not at all responsive towards the expectations of smaller Latin American countries. It pursued, instead, a rather intrusive policy in the region, for example, by intervening in support of a coup that brought to power a Brazil-friendly government in Bolivia in 1971, or by letting its secret services carry out ‘anti-subversive activities’ on Uruguayan territory (Hurrell, 1992: 42). While some elements of incoherence were in place — for example, the mixing of an aggressive ‘talk of “moving frontiers” and historical missions to regional predominance’, with a parallel rhetoric on the ‘need for Latin American unity’ (Hurrell, 1992: 27) — Brazil’s approach to the region during the 1960s–1970s was overall certainly not hesitant, and therefore not reluctant, but rather openly assertive.

Importantly, policies that are recalcitrant but not hesitant will not necessarily always be dominating and intrusive, like those of Brazil in the 1960s–1970s — they might also be the exact contrary. The previously mentioned cases of Japan and Germany, which consistently refused to resort to military power during the decades after the Second World War, are examples of a coherent, not hesitant — but, at the same time, recalcitrant — approach in the field of security.

The ‘grey zones’ of reluctance need to be interpreted particularly carefully. As was mentioned earlier, policies that are *moderately recalcitrant but extremely hesitant* belong to the grey zone if hesitation leads to paralysis, and they can shift to non-reluctance if inaction becomes consistent. The case of the EU, which can be considered a peculiar emerging actor, and its approach to the Libya crisis of 2011 provides a good illustration. In fact, the EU was extremely hesitant in its approach to the crisis. Long after the beginning of military operations by French, US, British and other forces, and ‘[w]ith [the North Atlantic Treaty Organization’s [NATO’s]] “Operation Unified Protector” well under way, the EU continued its debate as to whether it would have any military role in the conflict’ (Engberg, 2014: 159). It took the European Council three weeks to decide that it would establish a humanitarian mission to Libya, and the actual existence of that mission was made conditional upon a request by the UN Office for the Coordination of Humanitarian Affairs (OCHA) (Koenig, 2014: 259). As no such request was ever made, the EU mission, which had been prematurely called ‘EUFOR Libya’, never came into existence. The EU’s strong hesitation (lack of initiative and flip-flopping in the form of half-hearted measures combined with rhetorical condemnation)^13^ was paired with a lack of full responsiveness to ‘internal and external demands for [it] to act as a “comprehensive power”’ (Koenig, 2014: 251), mainly due to the divergences among the interests of some member states and European institutions (Engberg, 2014: 160–161; Koenig, 2014: 260–264). We can interpret the EU’s policies and its inability to make a decision on the establishment of a CSDP (Common Security and Defence Policy) mission as extreme hesitation and almost paralysis — a ‘grey zone’ given the potential for a transition from reluctance to consistent (and non-reluctant) rejection of any kind of engagement. Ultimately, however, the EU’s policies shifted back from the grey zone to reluctance: hesitation was not so extreme as to lead to complete inaction since a decision on the mission was eventually made; moreover, the fact that EUFOR Libya never came into existence was ultimately due not to the EU’s inaction, but to the lack of a request by OCHA.

If we move on to typical cases of reluctance, which do not involve the extremes of the continuum or grey zones, we will find a combination of *both hesitation and recalcitrance*. A good illustration is provided by India’s approach to crisis management in South Asia, which since the 1990s has been particularly reluctant. In post-2001 Afghanistan, for example, New Delhi has been highly engaged as a donor, but it has been hesitant on matters of security, despite the potentially detrimental consequences of a further destabilization of Afghanistan for India’s own security. India delayed and watered down the implementation of a strategic partnership agreement signed with Afghanistan in 2011 (Destradi, 2014: 569–570). On the issue of weapons provision to the Afghan National Security Forces (ANSF), New Delhi has been flip-flopping, initially refusing to help, later agreeing to supply some Indian-made light helicopters and logistics equipment, subsequently agreeing to provide weapons, but ultimately choosing to pay for Russian weapons deliveries (Miglani, 2014). India’s recalcitrance becomes evident as New Delhi repeatedly refused to comply with the Afghan government’s wishes for more support, thereby disappointing Afghan policymakers and observers (Destradi, 2014: 570).

A further useful illustrative case of reluctance is that of Germany in the Eurozone crisis — a case in which the notion of reluctance has been used to describe Germany’s attitude (Paterson, 2011; *The Economist*, 2013), but without an extensive discussion of what the term entails. The focus on the two constitutive dimensions of reluctance — hesitation and recalcitrance — allows us to notice some important shifts in Germany’s approach to the crisis over time. In fact, *recalcitrance* was in place throughout the whole crisis, from its very beginning in autumn 2009, when the German government labelled the crisis a Greek problem and refused to help, through to rejecting all the subsequent requests for help. This does not imply that Berlin stood aloof and ignored all requests since it was heavily involved in crisis management throughout, but it kept rejecting those demands and proposals that contradicted its preferences. Germany’s recalcitrance on Eurobonds, for example, remained in place over time, even though it was articulated in an increasingly harsh tone (*Spiegel Online*, 2012). By contrast, when it comes to *hesitation*, we can observe an interesting shift in Berlin’s approach. At the beginning of the crisis, the German government was highly hesitant: it did not take the initiative in crisis management; it delayed its responses to the crisis — among other reasons, in order not to alienate voters ahead of local elections in North-Rhine Westphalia in May 2010 (Schild, 2013: 28); and its approach was full of contradictions (flip-flopping). For example, Berlin had to backtrack on previous reassurances that it would not commit itself too heavily, and the contradictory statements issued by Finance Minister Schäuble and Chancellor Merkel in the early stages of the crisis gave ‘the impression that in Berlin the right hand didn’t know what the left was doing’ (Feldenkirchen et al., 2010: 22). Over time, however, German hesitation disappeared as Berlin took the initiative more openly, while delaying and flip-flopping diminished. Germany started promoting a policy of austerity in much more assertive and consistent terms, and with the European Council meeting of 8–9 December 2011 and the introduction of the Fiscal Compact (Schild, 2013: 37), German hesitation (and thereby its reluctance) had definitely disappeared.

In a contrary development, Brazil, which was not reluctant on matters of security and crisis management during the 1990s–2000s, has become increasingly passive and reluctant in recent years. Brazil’s new reluctance is exemplified by its approach to the crisis that shook Venezuela in 2014. While the government of Nicolás Maduro cracked down heavily on protesters, Brazil did not take any initiatives beyond participating in a failed mediation effort by UNASUR and was ‘characteristically mute’ (*The Economist*, 2014). Brazil’s hesitation was paired with recalcitrance about getting engaged more actively and was met with disillusionments among South American states that had hoped for more than some ‘bland’ communiqués on the part of Brasilia (Stünkel, 2014).

## Conclusion

This article has aimed to develop a conceptualization of reluctance in international politics. The conceptualization built upon: (1) existing uses of the concept in the literature in order to identify the broad semantic field to which it relates; and (2) the concept’s relationship to similar terms in the academic debate. Based on this concept reconstruction, reluctance was characterized as entailing the two constitutive dimensions of hesitation and recalcitrance. Corresponding indicators for empirical analyses were identified and the concept structure was outlined. The discussion of exceptionalism, isolationism, under-aggression and under-balancing has shown that the field of IR offers a range of conceptual tools related to reluctance, but that none of them is able to capture the essence of reluctance. The introduction of reluctance to the conceptual lexicon of IR has a value added in that it allows us to capture a particular way or style of foreign policy — one entailing hesitation and, at the same time, a recalcitrant attitude vis-a-vis the expectations articulated by others. This specific type of foreign policy seems to be rather widespread, but IR has so far failed to acknowledge its existence, usually assuming that states follow a clear and consistent foreign-policy path — or highlighting very specific forms of deviation from expected behaviour (as with under-aggression or under-balancing). The application of the concept of reluctance to different cases of rising powers has provided a first illustration of the usefulness of this concept: it allows us to analytically grasp a set of policies that are frequently mentioned in political commentary but never rigorously assessed. For example, a thorough assessment of specific indicators of hesitation and recalcitrance allows us to trace subtle changes in a country’s reluctance over time: in 2013, it did not make much sense anymore to consider Germany a reluctant power (*The Economist*, 2013) as, by that time, the German government had consolidated its position on the Eurozone crisis and had started acting in an openly assertive manner. The conceptualization of reluctance therefore fills a gap in the IR literature by giving us the analytical tools necessary to study a widespread type of foreign policy and by laying the groundwork for more extensive studies on reluctance, its determinants and its consequences.

The discussion of illustrative negative cases, in which only one of the secondary dimensions of reluctance was in place, has shown that both hesitation and recalcitrance are necessary conditions for a foreign policy to be considered reluctant. The case of Brazil, which in the 1990s–2000s was reluctant on regional integration but not on security matters, reveals that reluctance (or the degree thereof) can vary across issue areas. The notion of reluctance is therefore not tied to a specific policy field and does not necessarily just refer to an unwillingness to lead or to provide public goods, as is sometimes assumed in the literature on ‘reluctant hegemons’. Rather, reluctance is a more general approach to policymaking that can be identified across policy fields, periods of time and world regions.

Based on these insights, further research will need to study the determinants of reluctant foreign policy. In other words, why are rising powers reluctant and how can we explain variations in reluctance? Is this a phenomenon specifically related to rising powers and possibly associated to the difficulties and adjustment processes that these countries might face while suddenly getting more powerful? Furthermore, related to these points: how can we explain changes and shifts in the intensity of reluctance or between reluctance and non-reluctance? The assessment of the German case has revealed that as Berlin increasingly consolidated its position on the Eurozone crisis, it became less and less reluctant. Interestingly, this new determination could later also be observed in the field of security, with Germany’s proactive role in the management of the Ukraine crisis during 2014–2015. Similarly, India’s foreign policy under the government of Prime Minister Modi has arguably become less reluctant (Mohan, 2015). The contrast between the decreasing reluctance of India and Germany and the increasing reluctance of Brazil seems to suggest that reluctance might be a typical feature of rising powers’ foreign policy, which is left aside as countries get more powerful or are increasingly perceived as being more powerful, but that reluctance might re-emerge in the case of domestic instability like the one plaguing Brazil in recent years. More detailed assessments of the drivers of change in reluctant foreign policy, as well as of the relationship between ‘rise’ and reluctance, will be important areas of further research. Future studies would also need to include current and historical cases of declining powers. For example, an analysis of the recent non-reluctant policies of Russia would be useful for reaching more general conclusions about the rise and fall trajectories of great powers.

Moreover, the question emerges of what the broader potential impact of rising powers’ reluctant policies is. In fact, these countries’ reluctance does not seem to be confined to the management of regional crises like those discussed earlier. Reluctance is arguably also a core feature in rising powers’ approaches to broader processes of global governance. Several of these countries have repeatedly shown a lack of responsiveness vis-a-vis established powers’ requests for specific contributions to global governance, for example, on binding emission targets in global climate governance. However, they have not entirely opted out from global-governance mechanisms and institutions, but rather combined their recalcitrance with some degree of hesitation on how to contribute to the maintenance of international order. The concept of reluctance can therefore provide a useful analytical angle to scholars who try to make sense of broader processes of the allocation of responsibilities between established and emerging powers. While ‘reluctant guardians’ (Bunde and Oroz, 2015) might be contributing to a disruption of international order, more work is needed on whether the consequences of reluctance are, indeed, disruptive for global governance and on what policies might be needed to cope with reluctance and to address the concerns of reluctant rising powers.

## Notes

1. While Sartori clearly displays a greater interest in semantics as compared to other authors, as is revealed by his somewhat confusing discussion, for example, of the impact of translations (Goertz, 2006: 3), he clearly distinguishes between concepts and the words chosen to express them (Sartori, 1984: 18–22).

2. The analysis proceeded in a ‘data-driven way’, that is, by ‘letting [the] categories emerge from the data’ (Schreier, 2012: 25). The text corpus was analysed by looking specifically for expressions associated with the notion of reluctance or used to describe behaviour defined as ‘reluctant’. This approach is suitable given the descriptive aim of the exercise (Schreier, 2012: 43): the identification of the broader semantic field of concepts that are associated with reluctance in the literature. The different aspects emerging inductively from the text were summarized in categories, which are highlighted in italics in this article: ambivalent attitude, hesitant behaviour, selective commitment, incoherence, obstructionism towards others’ initiatives and slowness. Figure 1 visualizes the relationship between more specific aspects, such as ‘decline financial contributions’ or ‘incomplete implementation’, and the inductively elaborated categories, which are visualized in bigger characters as emerging out of the concept cloud.

3. On a rather different note, Ikenberry (1998/1999: 63) argues that the US acted as a ‘reluctant superpower’ in the post-war period since ‘it was not eager to actively organize and run’ the international order that it had created. While this underscores the selectivity of commitment highlighted by other authors, Ikenberry interestingly argues that this reluctance had a reassuring effect on other states.

4. A separate, but mostly prescriptive, strand in the literature on US foreign policy revolves around the notion of restraint (or self-restraint), understood as a moderate and subtle policy focused on few threats and thereby opposed to ‘expansiveness’ (Posen, 2014: xiii) and interested, instead, in reassuring others about one’s benign intentions (e.g. Ikenberry, 1998/1999). While the concept of restraint is clearly related to the four concepts discussed here, it is conceptualized as a straightforward policy that does not entail the elements of hesitation and recalcitrance typical of reluctance.

5. At the same time, isolationism is closely related to and sometimes equated with exceptionalism. As was mentioned earlier, the borders between the four concepts are blurred and these notions are sometimes overlapping.

6. Goertz (2006: 35) argues that the secondary-level dimensions of a concept will ‘almost always refer to the positive concept’ and that only in a second step will we have to think about the negative pole of each secondary-level dimension. In this case, however, I proceeded inversely by first thinking about what the constitutive dimensions of reluctance are not before moving on to define them. This approach is extremely helpful in order to clarify the secondary dimensions themselves.

7. Specific thresholds can be set in single empirical studies depending on the issue area and the subject.

8. This indicator is not limited to a specific cause of the delay, which can be either purposeful, that is, employed as part of a bargaining strategy, or unintended, for example, due to specific procedures or obstacles in the decision-making process. This reflects the fact that reluctance can be the result of either an unwillingness or an inability to pursue a determined and responsive policy. As acknowledged by Goertz (2006), causal thoughts are always inherent in the process of concept formation.

9. The notion of flip-flopping has been developed in the literature on party politics and elections (see, e.g., Burden, 2004: 214–215; Tavits, 2007: 154–155). Of course, it is always difficult to distinguish frequently changing policies from a normal process of adaptation related to learning effects or to changed circumstances, which can, ex post, easily be confused with hesitation (I am grateful to Michael Brzoska for highlighting this aspect to me). If policies keep changing back and forth instead of developing in a linear manner, however, we can conclude that this will be an indicator of reluctance rather than of learning. In any case, the inclusion of indicators for recalcitrance, which must be in place as well, helps in assessing reluctance.

10. According to Goertz (2006: 42), ‘there are no formal reasons opposing continuous dimensions for either the necessary and sufficient condition or the family resemblance concept structures’. While a necessary-and-sufficient-conditions structure is usually associated with a dichotomous view of categories, it is, indeed, possible to ‘separate the issue of dichotomous concepts from their structure’ (Goertz, 2006: 29).

11. Goertz points out that grey zones exist in both family resemblance and necessary-and-sufficient-conditions concept structures, but are smaller in the latter (see the discussion of measurement consistency and the use of the minimum operator (Goertz, 2006: 120)).

12. Interestingly, the growing body of literature on ‘rising’ or ‘emerging’ powers is characterized by a dearth of precise operationalizations. Besides power resources and regional preponderance, many studies also include the criterion of aspiration to a greater role in world politics (e.g. Schirm, 2010: 198) or the peculiar negotiating position of rising powers: they are veto players that have not yet developed into agenda setters (Narlikar, 2013: 561–562).

13. See, for example, European Council (2011).
